# Novel Water–Oil Mixed Frying: Fried Oil Quality and the Formation of Heterocyclic Amines and *Trans* Fatty Acids in Fried Duck

**DOI:** 10.3390/foods11050626

**Published:** 2022-02-22

**Authors:** Muneer Ahmed Jamali, Zhen Wang, Yuxia Zhu, Yawei Zhang

**Affiliations:** 1 National Center of Meat Quality and Safety Control, College of Food Science and Technology, Nanjing Agricultural University, Nanjing 210095, China; majamali65@yahoo.com (M.A.J.); 2016108046@njau.edu.cn (Z.W.); 2 School of Biological Science and Food Engineering, Chuzhou University, Chuzhou 239004, China; 2016208019@njau.edu.cn

**Keywords:** water–oil mixed frying, harmful substances, meat quality, oil quality, cooking

## Abstract

The present study was conducted to explore the impact of novel water–oil mixed frying and traditional oil frying methods on the soybean oil quality and formation of *trans* fatty acids (TFAs) and heterocyclic amines (HCAs) in fried duck breast and skin during 60 frying cycles. The acid value of the soybean oil was 2.10 mg/g using the traditional oil frying and 1.08 mg/g using water–oil mixed frying at the 60th frying cycle. The peroxide value of the water–oil mixed fried soybean oil was significantly lower than that of the traditional frying method. Water–oil mixed frying delayed the formation of TFAs in the soybean oil. The traditionally oil fried skin showed increased TFAs (9*t*C16:1) content from 0.17 to 0.22 mg/g (29.4% increase), while those of the water–oil mixed fried samples increased from 0.16 to 0.20 mg/g (25.00% increase) compared to control. Harman, Norharman and AαC were detected in all the fried samples and PhIP was observed after the 20th frying cycle, while MeAαC was formed only at the 60th frying cycle in traditional fried duck breast. Therefore, it is concluded that the water–oil mixed frying method is more suitable to improve the quality and safety of fried duck skin and breast meat.

## 1. Introduction

The manufacturing of meat products relies on several processing methods, including frying, and conventional frying oil temperatures fall in the range of about 150–190 °C [1]. The most common prevailing drawback of frying protein-rich meat at these temperature ranges is the formation of mutagenic and carcinogenic compounds such as heterocyclic amines (HCAs) [2]. Until now, 30 kinds of HCAs have been identified from different sources [3] and most of these HCAs have been previously identified as human health hazards. The International Agency for Research on Cancer (IARC) has recognized nine HCAs, including 2-amino-3,4-dimethyl-imidazo [4,5-f]quinoline (MeIQ) (group 2B), 2-amino-1-methyl-6-phenylimidazo[4,5-b]pyridine (PhIP), 2-amino-3-methyl-9H-pyrido[2,3-b]indole (MeAαC) and 2-amino-9H-pyrido[2,3-b]indole (AαC) as possible human carcinogens, and IQ (2-amino-3-methylimidazo[4,5-f]quinoline) as a probable human carcinogen. Based on their carcinogenic nature, IARC has suggested that dietary intake of these HCAs should be minimized [4,5], while the specific intake of HCAs likely to cause cancer in humans has not been determined. Among various factors contributing to the formation of HCAs, fried oil is also a key factor which affects the contents and types of HCAs. The repeated use of fried oil results in the further accumulation of HCAs in the food products. Several reports have shown the formation of HCAs may be the result of oil oxidation during frying and the repeated use of the same frying oil [2,6]. It has been observed that during frying, a number of nutrients in the meat can move to the meat surface along with water, which later on results in the evaporation of the water, during which some protein, free amino acids, creatine and organic acids may be accumulated in the high-temperature fried oil. This accumulation could provide favorable conditions for the occurrence of the Maillard reaction, resulting in the deterioration of fried oil color and the formation of HCAs.

Considering the facts about HCAs formation, several approaches have been adopted to mitigate the generation of these HCAs, but most of these strategies focused on the usage of phenolic compounds of natural antioxidants in order to control the generation of HCAs in meat products [7]. On the other hand, frying at a temperature above 150 °C can also generate *trans* fatty acids (TFAs) that can elevate the cholesterol levels in blood and is related to the risk of coronary heart disease. It is a well-documented fact that TFAs are usually formed at high temperatures during oil frying of meat products or the prolonged heating time [2,8]. However, limited information is available regarding the TFAs profile in meat products with repeated use of the same frying oil (repeat cycling). Therefore, it is of dire need to explore the TFAs profile in repeatedly used frying oil because there is a mass transfer of TFAs between the oil and meat components during the frying [9,10]. It is of utmost importance to adopt an alternative way of frying to reduce the generation of mutagenic and carcinogenic compounds in meat products. 

Frying is a common method to transfer the heat for the cooking of food products [11]. During frying, oil type, time, temperature and fryer type affect the quality of oil, due to polymerization, oxidation and hydrolysis [12]. Recently, the water–oil mixed frying method was developed which contained a mixture of water and oil during frying. The water–oil mixed frying method has considerably several advantages over the traditional method [13]. During water–oil frying, oil remains in the upper layer and water can reside within its sublayer and the frying residues can accumulate within the water layer before they are carbonized in the oil layer [14]. Hypothetically, this way will greatly reduce the degree of fried oil deterioration and harmful compound generation, since repeat frying cycles and prolonged heating time causes several chemical reactions which lead to the formation of human health hazard substances in oil and meat products [14]. Nevertheless, the effect of the water–oil mixed frying method on the formation of TFAs in the fried oil and the generation of HCAs and TFAs in the meat products is not yet known. Therefore, in the current study, the outcomes of the traditional oil frying method and the water–oil mixed frying method on the formation of HCAs and TFAs in duck breast skin/meat and frying oil quality were evaluated during 60 frying cycles. The generated data will provide the baseline information about the novel water–oil mixed frying method on the quality of fried meat products to minimize the formation and accumulation of harmful compounds in the fried meat products.

## 2. Materials and Methods

### 2.1. Chemicals

HCAs standards including, 2-amino-3,8-dimethylimidazo[4,5-f]quinoxaline (MeIQx), 2-amino-3,4-dimethylimidazo[4,5-f]quinoline (MeIQ), 2-amino-3-methylimidazo[4,5-f]quinoline (IQ), 2-amino-3,7,8-trimethylimidazo[4,5-f]quinoxaline (7,8-DiMeIQx), 2-amino-3,4,8-trimethylimidazo[4,5-f]quinoxaline (4,8-DiMeIQx), 1-methyl-9H-pyrido[3,4-b]indole (Harman), 9H- Pyrido[4,3-b]indole (Norharman), 3-amino-1,4-Dimethyl-5H-pyrido[3,4-b]indole (Trp-P-1), 3-amino-1-methyl-5H-pyrido[4,3-b]indole (Trp-P-2), 2-amino-1-methyl-6-phenyl-Imidazo[4,5-b]pyridine (PhIP), 2-Amino-3-methyl-9H-pyrido[2,3-b]indole (MeAαC) and 2-amino-9H-pyrido[2,3-b]indole (AαC) were obtained from Toronto Research Chemicals (Downsview, ON, Canada). *Trans* fatty acid methyl ester (FAME) standards including, 9 t-14:1, 9 t-16:1, 6 t-18:1, 9 t-18:1, 11 t-18:1, 9 t,12 t-18:2, 11 t-20:1 and 13 t-22:1 were purchased from Nu-Chek Prep, Inc. (Elysian, MN, USA). The HPLC-grade solvents acetonitrile and methanol were bought from Tedia Co. (Fairfield, OH, USA), while ammonia, ammonium acetate, hydrochloric acid and sodium hydroxide were collected from Sinopharm Chemical Regent Co., Ltd. (Shanghai, China). All reagents and chemicals were chromatographic or analytical grade. A Millipore water purification system was used to collect the deionized water (Millipore Co., Bedford, MA, USA).

### 2.2. Samples and Frying Method

Frozen duck breast samples with skin (6.1 mean pH value) were bought from a local meat market in Nanjing, PR China and thawed at 4 °C for 12 h before use. All the samples were divided into two groups to process using two different frying methods, i.e., traditional oil frying and water–oil mixed frying, and 60 frying cycles were completed for each group. Each frying cycle contained three raw breast muscles, while the three raw samples without frying were analyzed and presented as (0 frying cycle). As samples for the frying, soybean oil was poured into a water–oil mixed fryer (Expro Machinery Engineering Co., Ltd., Hangzhou, China) and the water was added up to the water line. The oil temperature was set at 170 °C, and three raw duck breasts of the same size (12 cm × 8 cm × 2 cm; 150 ± 5) were fried for 10 min. All the meat samples were without any seasoning to eliminate the influence of seasoning. Ten batches (frying cycles) were consecutively fried per day for six days. The fried oil was not changed, nor replenished with fresh oil during the process. The oil temperature remained at 170 ± 5 °C. Finally, samples were taken from the 1st, 10th, 20th, 30th, 40th, 50th and 60th frying cycle. The skin and meat of the duck breast samples were separated and individually grinded with a tissue homogenizer (8010S, Waring, Inc., Torrington, CT, USA), vacuum packed, and then frozen at −80 °C until testing. On the other hand, 50 mL of oil samples were collected after frying was completed each day and frozen at −80 °C until analysis. The same operation process was conducted for the traditional oil frying method using a fryer (EF101-V, Changzhou Guohua, China). 

### 2.3. Determination of Color, Peroxide Value and Acid Value of the Soybean Oil

The color of oil samples was determined by spectrophotometer method. Briefly, 2 mL of the filtered oil was added into 6 mL of petroleum ether and then boiled at a range of 60–90 °C. After boiling samples were vortexed and the absorbance of the sample was measured at 880 nm, petroleum ether was used as a blank. The acid and peroxide values of the soybean oil samples were determined according to the measuring method of GB/T5009.37-2003, the national standard of the People’s Republic of China, methods for analysis of hygienic standards of edible oils. 

### 2.4. Determination of Color Difference (∆E) and Moisture Content of Duck Meat and Skin

The moisture percentage was analyzed according to the method reported in GB5009.3-2016, Chinese National Standard (Determination of water in foods). For determination of the differences in the color (∆E), all the samples were placed in a strainer to drain the excess oil and skin was removed manually from the breast meat with the help of a knife. After 0.5 h, a colorimeter (CR-400, Konica Minolta Sensing, Inc., Osaka, Japan, 10° standard observer and D65 illuminant) was used to measure the L* (lightness), a* (redness) and b* (yellowness) values at six randomly selected locations of the samples and means values of these readings were used for further analysis. The instrument was calibrated using a white reference plate. The color difference (∆E) was calculated according to the following formula [15].

(1) $$∆E=\sqrt{{\left(∆L^{*}\right)}^{2}+{\left(∆a^{*}\right)}^{2}+{\left(∆b^{*}\right)}^{2}}$$

### 2.5. Determination of Trans Fatty Acids (TFAs)

A gas chromatography (GC-2010Plus Gas Chromatograph Shimadzu Corporation, Kyoto, Japan) was used to analyze the TFAs profile. After fat extraction, KOH in isooctane and methanol was used to prepare the methyl esters. The trans FAMEs were analyzed according to the method described in the Chinese National Standard for the determination of *trans* fatty acids in foods with some modifications [2]. An SP-2560 column (L × I.D. 100 m × 0.25 mm, df 0.20 μm) (Supelco, Centre County, PA, USA) was used. The temperature was maintained at 140 °C for 5 min, then rose to 200 °C at a rate of 2 °C/min, and then increased to 208 °C at 1 °C/min and kept there for 15 min. Nitrogen gas was used as a carrier gas. The other conditions reported included flow rate (1.3 mL/min), injection volume (1 μL) and split ratio (30:1). The concentration of the standard substances ranged between 1–500 mg/L, the linear correlation coefficient was within 0.9997–0.9999, and the recovery rate of standards was 92.05–98.54%. Moreover, the similar structures and close retention times of three *t*C18:1 isomers (6*t*C18:1, 9*t*C18:1, 11*t*C18:1) resulted in overlapping peaks which made it difficult to separate completely, hence these three isomers are considered as one substance (*t*C18:1).

### 2.6. Analysis of Heterocyclic Amines

The extraction and purification of HCAs were determined by the procedure reported by Gross and Grüter [16] with some modifications. Determination and quantification were achieved as reported in the previous studies of our laboratory [17,18] using HPLC (Waters 2695 High Performance Liquid Chromatograph Waters Company, Worcester County, MA, USA). Separation was performed using a TSK gel ODS-80 TM column reversed-phase (25 cm, 4.6 mm, 5 µm, 80 Å, Tosoh, Tokyo, Japan). A vacuum manifold was used for solid-phase extraction (CNW Technologies GmbH, Düsseldorf, Germany). The column temperature was set to 30 °C; the flow rate was 1 mL/min and the injection volume per injection was 20 µL. The 12 types of HCAs were detected through an ultraviolet detector (IQ, MeIQx, MeIQ, 4,8-DiMeIQx and 7,8-DiMeIQx) and fluorescence detector (Harman, Norharman, Trp-P-1, Trp-P-2, PhIP, MeAαC and AαC). Using methanol as a solvent, the polar HCAs were diluted to 1, 10, 50, 100, 250, 500, 1000 ng/mL mixed standard solution; the non-polar HCAs were diluted to 0.1, 1, 2, 10, 20, 50, 100 ng/mL mixed standard solution to obtain a standard curve. The linear correlation coefficients of heterocyclic amines were between 0.9972 and 0.9999. The linear ranges of IQ types and non-IQ types of HCAs were 10–1000 ng/g and 0.1–100 ng/g, respectively. The recovery rates of 12 kinds of HCAs were 67.12–96.81%.

### 2.7. Statistical Analysis

Minitab 18.1 statistical software was used to perform the statistical analysis. The data was tabulated and presented as the mean of replicates and standard deviation. Data was further analyzed using two-way ANOVA and Fisher’s LSD test on the interaction of the two factors at *p* < 0.05. All the experiments were repeated thrice.

## 3. Results and Discussion

### 3.1. Color, Acid Value and Peroxide Value of the Soybean Oil

**Figure 1:**
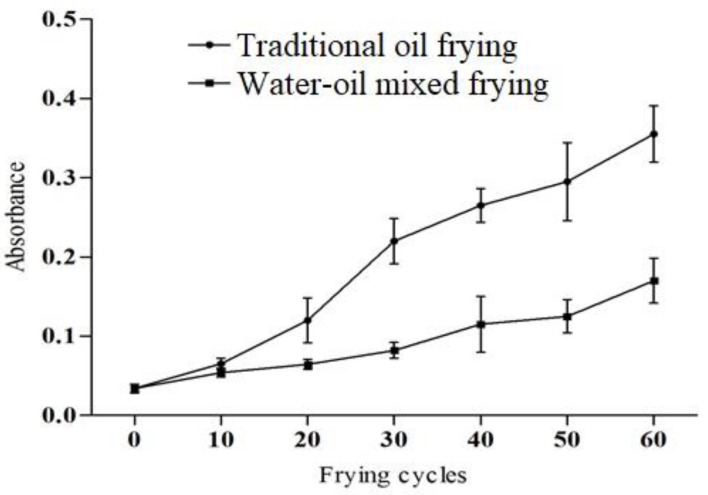
The color of soybean oil under different frying methods and frying cycles.

The soybean oil color change corresponding to the frying methods and frying cycles is shown in Figure 1. The absorbance of the samples fried with the water–oil mixed frying method is lower than that of the traditional frying method. In the previous studies, it is reported that the darkening of the color might be due to the availability of non-volatile degradation products including oxidized triglycerides and polymers [19,20]. Interaction of food nutrients also causes Maillard reaction and browning products contributes to the darkening [21]. 

**Figure 2:**
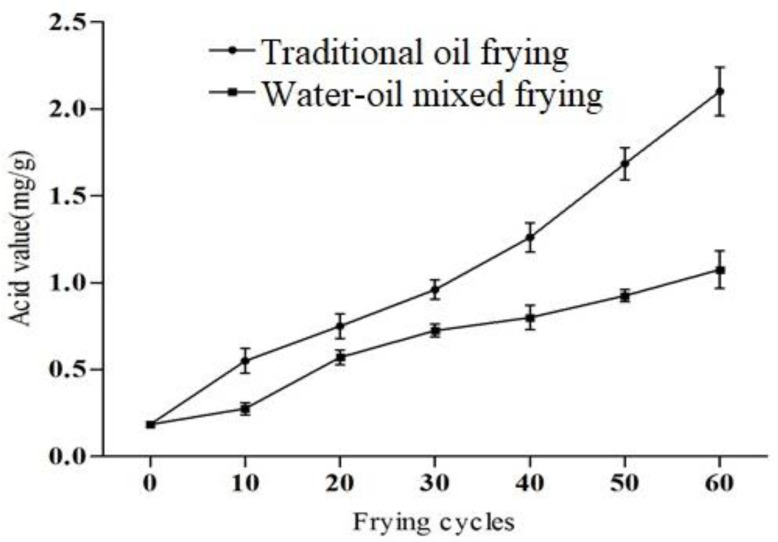
The acid value of soybean oil under different frying methods and frying cycles.

Oils and fats are hydrolyzed at high temperatures to produce free fatty acids, this in turn accelerates the rancidification, which is primarily determined by acid value [22]. The acid value of fried soybean oil was increased considerably with the number of frying cycles in both frying methods (*p* < 0.05). Figure 2 shows that the increased acid value was faster under traditional oil frying conditions than the water–oil mixed frying method during 60 frying cycles. The acid value of the oil was 2.10 mg/g when using the former method and 1.08 mg/g when using the latter method (11.05- and 5.68-fold greater, respectively, than the original content (0.19 mg/g). None of the acid values in this work exceeded the 3 mg/g limit specified in the GB2716-2005 protocol. Moreover, observed findings for acid value is also as per recommendation (<3) by the Food Sanitation Act, Japan [23]. After 40 frying cycles, the acid value of the traditional fried soybean oil was significantly higher than the water–oil mixed frying and this difference between the two values gradually expanded with the increasing frying cycles. A similar increasing trend was recorded in the previous study; the acid value of the soybean oil was increased from 0.47 to 5.14 after 1–101 frying cycles [24]. During processing in the frying oil, the acid value was increased maybe due to oxygen, steam and water initiated chemical reactions [25]. Furthermore, chemical composition of fats and oils also have a role in oxidative changes [24].

**Figure 3:**
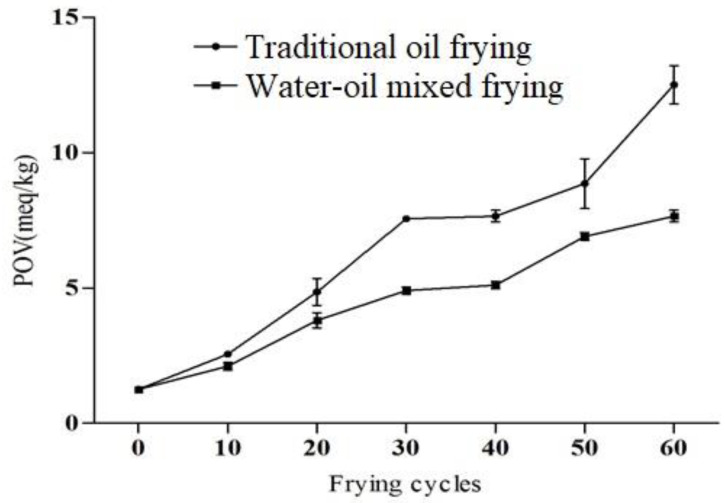
Peroxide value (POV) of soybean oil under different frying methods and frying cycles.

The peroxide value of oils is an important factor used to measure the degree of oxidative rancidity [24]. The peroxide value of fresh oil should be <1 meq/kg, and if >10 meq/kg, the fats and oils are considered as rancid [26]. In our study, the peroxide value was increased significantly during 60 frying cycles, the peroxide value of traditional fried soybean oil and water–oil mixed frying were 12.50 meq/kg and 7.65 meq/kg, respectively, after 60 frying cycles (Figure 3), though this value of fresh soybean oil was only 1.25 meq/kg. However, its peroxide value still met the GB2716-2005 limit (25 meq/kg). In addition, Sulieman et al. [27] also recommended that a good quality frying oil should have <2 meq/kg peroxide value. After 30 cycles, the effects of both frying methods on the peroxide value of the soybean oil was found to be considerable (*p* < 0.05), and the peroxide value of the water–oil mixed fried oil was significantly lower than that of the traditional fried soybean oil. Meat residue readily fell into the water layer during frying with the water-oil mixed fryer. Hence this method reduces the accumulation and carbonization of residues in the oil, subsequently decreasing the oil turbidity and deterioration [13]. Ma et al. [14] also revealed that water–oil mixed frying reduced the deterioration of the soybean oil. Due to the above fact, in the current study water–oil mixed frying might reduce the oxidation of the oil and delay the increase in the peroxide value. Similar trends of an increase in peroxide value in oils during continued frying for two consecutive days were reported earlier [28,29]. Furthermore, oxidation of the oils and fats depends on various factors, such as heating rate, time, temperature, light, oxygen concentration, storage conditions and composition of fatty acids [30]. Based on the above indicators, it can be concluded that in the repeated frying cycles of soybean oil, the quality of the oil continues to deteriorate, and water–oil mixed frying can delay the rate of deterioration of the oil.

### 3.2. TFAs Contents in the Soybean Oil

The *cis* double bonds in unsaturated fatty acids are isomerized upon prolonged heating to give TFAs [8]. In the current study, only *t*C18:1 was detected in the fried soybean oil, and it was found that the *t*C18:1 content in the raw soybean oil was 1.28 mg/g, which is comparable with the findings of Wang et al. [2]. The content of *t*C18:1 in the oil showed an increasing trend as the oil was repeatedly reused (Table 1). The *t*C18:1 content of the frying oil after 60 cycles was 1.52- and 1.43-fold increased compared to the initial content of the fresh oil for both frying methods, traditional and water–oil mixed frying, respectively. Water–oil mixed frying delayed the formation of TFAs in the soybean oil. In the present work, after 20 cycles of the oil, the amount of *t*C18:1 produced in the soybean oil during water–oil mixed frying was significantly lower than that produced by the traditional frying method, the *t*C18:1 content in soybean oil after 60 cycles in water–oil mixed frying was comparable to the *t*C18:1 content in soybean oil after 40 cycles in the traditional oil frying method. In addition, the formation of 9*t*C16:1 (0.17 mg/g) was detected in soybean oil after 60 cycles of traditional frying (data not shown). Correspondingly, in a previous study initially TFAs were not observed in soybean oil samples, while they were detected after 70 h of frying [31]. In another study, at 170 and 180 °C no obvious difference was observed between trans isomers of soybean oil, whereas trans isomers were increased at 190 °C in soybean oil [32]. Jain et al. [33] also stated that TFAs in oils were increased with the increase in frying cycles and temperatures. Dana et al. [34] continuously injected water droplets into the bottom of continuously heated canola oil, which helped prevent changes in the fatty acid composition of the oil, as compared to the control group. The inhibition of TFAs production in frying oil by mixing it with water may be due to meat residue falling into the water layer during frying, thus reducing the introduction of oxidation catalysts, such as iron ions in the meat, and delaying the oxidation of the oil. 

### 3.3. Color Change (∆E) and Moisture Percentage in the Fried Duck Breast Skin and Meat

Table 2 shows that changes in the color (∆E) of the traditionally oil fried duck breast skin increased significantly with the number of frying cycles, the ∆E of the fried skin of samples collected from the 60th cycle were significantly greater than those of the 1st and 10th frying cycles in traditional oil frying. Whereas, in the samples fried with the water–oil mixed frying, the ∆E of the fried duck breast samples was relatively stable, with no significant differences between the samples taken from the 1st and 60th cycle. There was no significant difference between the ∆E values of the duck breast meat fried with the water–oil mixed fryer and the traditional oil fryer. Ma et al. [14] reported that in the water–oil mixed frying method, a decreasing rate of lightness during the six days frying as compared to the pure oil frying was observed, while yellowness was increased in both methods during frying. Srivastava and Semwal [35] suggested that higher yellowness was associated with the polymer formation of unsaturated carbonyl compounds and non-polar compounds of food stuff solubilized in the oil. The moisture contents of the fried duck meat samples were significantly lower than those of the fresh samples, and these results are in line with previous study [2]. It was revealed that the moisture contents of the fried skin samples decreased with the increase in frying cycles regardless of frying methods. The moisture content of the fried skin sample from the 60th cycle was significantly lower than that of the fried skin sample from the 1st cycle in traditional oil frying. This may be caused by the increase in the impurities and viscosity of the fat over extended periods of use, resulting in reduced lipid exchange with the duck skin and subsequently increased amounts of fat retained in the skin (data not shown). The moisture content of the fried duck breast meat was not remarkably affected by the number of frying cycles nor the frying method (except at the 20th and 50th cycles), which might be due to the dry and hard crust that was rapidly formed during frying which prevented further evaporation of moisture.

### 3.4. TFAs Contents in the Fried Duck Breast Skin and Meat

Table 3 shows the variations in the TFAs contents in the skin and meat corresponding to the frying cycles and frying methods and the limit of detections for 9 t-14:1, 9 t-16:1, 9 t-18:1, 6 t 18:1, 11 t 18:1, 9 t,12 t-18:2, 11 t-20:1 and 13 t-22:1 were 0.063, 0.064, 0.068, 0.072, 0.068, 0.07, 0.05 and 0.12 mg/100 g, respectively. In the present study, only two types of TFAs, i.e., 9*t*C16:1 and *t*C18:1, were detected in fried samples, however 9*t*C16:1 was observed only in the skin of the fried duck and was not detected in fried breast duck meat in both frying methods. These results are in agreement with Liu et al. [36]; they stated that the formation of TFAs were observed in the chicken leg skin and no TFAs were formed in the chicken leg meat, which might be due to the absorption of oil in the skin. The content of 9*t*C16:1 in the fried duck skin of the traditional oil frying method sample at the 60th cycle was significantly greater than that of other frying cycles in the same method. Upon comparing the 9*t*C16:1 content in the fried skin of the samples of the 60th and 1st cycle, the traditionally oil fried samples showed increased TFAs contents from 0.17 to 0.22 mg/g (a 29.41% increase), while those of the water–oil fried samples had increased from 0.16 to 0.20 mg/g (a 25.00% increase). Similarly, *t*C18:1 content increased under both frying methods. The *t*C18:1 content in the samples after the 40th cycle was greater than the 1st to the 30th cycles when the traditional oil frying method was employed, whereas the *t*C18:1 content in the samples fried in the water–oil mixed fryer did not show significant increases until the 50th cycle. The two frying methods did not exhibit significant differences on the 9*t*C16:1 and *t*C18:1 content in the fried skin, except after the 40th frying cycle when the 9*t*C16:1 content in the fried skin of water–oil mixed fried samples was lower than that in the fried skin of traditionally oil fried samples. Similarly, for the *t*C18:1 content, no significant differences were noted in the samples fried under either condition, while a numerical uptrend was observed with increasing frying cycles. On the other hand, TFAs contents were lower in the fried breast duck meat samples as compared to the fried skin, *t*C18:1 being the only detected TFA in the fried duck breast in both processing methods. Lower TFAs content in the fried breast meat and higher TFAs in the fried skin shows that the absolute content of the TFAs intake in the samples related to the amount of TFAs in the frying oil. The content of *t*C18:1 increased with the increasing frying cycles. Yang et al. [37] concluded that the contents of TFAs in chicken fillets have no statistical variation among various frying cycles. Additionally, the concentration of TFAs also depends on the amount of TFAs present in the feed of poultry birds [38].

### 3.5. Heterocyclic Amines in the Fried Duck Breast Skin and Meat

The effects of frying cycles and frying methods on the HCAs content in the fried skin and duck breast meat are shown in Table 4. The limits of detections for harman, norharman, PhIP, Trp-P-1, Trp-P-2, MeAαC, AαC, IQ, MeIQ, MeIQx, 4,8-DiMeIQx and 7,8 DiMeIQx were 0.05, 0.07, 0.03, 0.04, 0.04, 0.02, 0.02, 2.95, 1.68, 0.91, 0.72 and 0.83 ng/mL, respectively. For the skin of the duck breast, the types and contents of HCAs increased with the number of frying cycles. Three HCAs, namely Harman, Norharman and AαC were found in all the fried samples after the first frying cycle. These results are in accordance with the previous study of Liao et al. [39]; they also found AαC, Harman and Norharman in all deep-fried duck breast meat. PhIP was detected at the 30th frying cycle. This might be due to the oxidation of oil and fat during repeated frying contributing to PhIP formation. The oxidation products of unsaturated fatty acids, especially 2,4-dienal and 2-enal, made a significant contribution to the production of PhIP [40], while other types of HCAs were not detected in any fried skin sample in both frying methods, which might be due to the fact that compounds such as 4,8-DiMeIQx, MeIQx, IQ and MeIQ come under the umbrella of thermic set of HCAs and generate at long cooking times and high temperatures. Moreover, the data shows that in the fried duck skin the water–oil mixed frying caused a lower content of Norharman than traditional oil frying at the 60th frying cycle (*p* < 0.05) and the amount of Norharman was also increased significantly with the increase in frying cycles. Guo et al. [41] also reported that Norharman content was increased in pig hock skin during four cooking cycles. Additionally, Randel et al. [42] conducted a study and observed the degradation of HCAs in oil under frying and storage conditions and reported that HCAs may be soluble in oil and Norharman had an oil–water partition coefficient (Ko/w) of 36.8 ± 3.5. Additionally, Harman and Norharman were more stable as compared to the other types of HCAs under similar conditions. The water–oil mixed frying method left oil in the upper and water in the lower layer, automatically filtrated oil sample scraps off and retarded the precursors accumulation in the oil [14], which would benefit the reduction of Norharman. Apart from the frying method, the amount and types of HCAs also depends on several other factors including pH level, availability of precursors, additives, water activity and the type of meat [43]. Furthermore, in our study, similar trends were noted in the case of fried duck breast meat as Harman, Norharman and AαC contents were detected in the 1st frying cycle in both processing methods and PhIP was detected in the samples from the 20th frying cycle. Compared to the fried duck skin, MeAαC was detected in the fried duck breast meat only under the traditional oil frying method. There was no statistical variation in the content of Harman and AαC at various frying cycles between water–oil mixed frying and the traditional oil frying method (*p* > 0.05). The oil temperature of the two frying methods was maintained at 170 ± 5 °C, and the samples were fried at the same oil temperature and in the same medium, so most of the data have no significant difference. Moreover, an increasing trend was observed in all types of detected HCAs with increasing frying cycles in both frying methods, possibly due to the generation of free radicals in the oil which makes promising conditions for the generation of HCAs [2].

## 4. Conclusions

The water–oil mixed frying method reduced the *trans* fatty acids, peroxide value and acid value of the soybean oil when compared to the traditional oil frying method during 60 frying cycles. The ∆E and moisture content of the fried duck skin and breast meat were also relatively stable using the water–oil mixed frying method. The content of TFAs were higher in the fried duck skin than fried duck breast meat in both processing methods. Harman, Norharman and AαC were formed in all fried duck breast and skin samples and PhIP was observed after the 20th frying cycle, whereas MeAαC was only formed at the 60th frying cycle in traditionally oil fried duck breast meat. Both TFAs and HCAs had a slightly increasing trend with the increase in frying cycles. Data shows that the novel water–oil mixed frying method is more suitable than traditional oil frying. Therefore, this method may be used at industrial as well as household cooking levels. These findings will be beneficial for the regulations regarding frying oils used for food processing. Furthermore, a public awareness drive and good manufacturing practices (GMPs) should be adopted to reduce the intake of HCAs and TFAs from processed meat. 

## Figures and Tables

**Table 1:** Contents of TFAs in the soybean oil under different frying methods and cycles.

Number of Cycles	*t*C18:1 (mg/g)	
	Traditional Oil Frying	Water–Oil Mixed Frying
0	1.28 ± 0.03 ^hi^	1.28 ± 0.03 ^hi^
10	1.36 ± 0.05 ^g^	1.32 ± 0.01 ^gh^
20	1.46 ± 0.05 ^f^	1.25 ± 0.02 ^i^
30	1.73 ± 0.05 ^d^	1.37 ± 0.02 ^g^
40	1.88 ± 0.05 ^bc^	1.46 ± 0.02 ^f^
50	1.90 ± 0.06 ^ab^	1.65 ± 0.04 ^e^
60	1.95 ± 0.06 ^a^	1.83 ± 0.05 ^c^

Note: Statistics are shown as mean ± standard deviation; means bearing different superscripts show significant difference at *p* < 0.05.

**Table 2:** ∆E and moisture percentage of duck skin and breast meat under different frying methods and cycles.

Number of Cycles	∆E (Duck Skin)		∆E (Duck Breast Meat)		Moisture (Duck Skin)		Moisture (Duck Breast Meat)	
	Traditional Oil Frying	Water–Oil Mixed Frying	Traditional Oil Frying	Water–Oil Mixed Frying	Traditional Oil Frying	Water–Oil Mixed frying	Traditional Oil Frying	Water–Oil Mixed Frying
0	0.00 ± 0.00 ^e^	0.00 ± 0.00 ^e^	0.00 ± 0.00 ^d^	0.00 ± 0.00 ^d^	23.15 ± 0.20 ^g^	24.04 ± 0.20 ^g^	72.98 ± 0.40 ^a^	74.06 ± 0.40 ^a^
1	23.81 ± 2.67 ^d^	26.00 ± 0.59 ^bcd^	13.19 ± 2.70 ^bc^	15.56 ± 0.90 ^ab^	37.01 ± 1.10 ^abc^	39.48 ± 0.11 ^a^	62.85 ± 0.04 ^defg^	61.63 ± 0.78 ^gh^
10	23.58 ± 3.14 ^d^	29.54 ± 2.17 ^ab^	12.77 ± 0.44 ^c^	13.89 ± 0.60 ^bc^	36.32 ± 0.39 ^bc^	34.80 ± 0.26 ^cde^	62.39 ± 0.31 ^efgh^	61.99 ± 0.48 ^fgh^
20	29.96 ± 1.32 ^a^	27.91 ± 2.86 ^abc^	13.68 ± 1.48 ^bc^	14.99 ± 0.92 ^abc^	35.50 ± 0.94 ^bcd^	35.94 ± 2.96 ^bcd^	64.01 ± 0.45 ^bcd^	62.41 ± 0.22 ^efgh^
30	24.57 ± 1.03 ^cd^	29.65 ± 0.45 ^a^	14.86 ± 3.12 ^abc^	14.94 ± 1.09 ^abc^	37.38 ± 0.03 ^ab^	32.44 ± 0.53 ^ef^	63.07 ± 0.38 ^cdef^	64.30 ± 0.22 ^bc^
40	26.98 ± 2.21 ^abcd^	30.15 ± 2.21 ^a^	12.76 ± 1.93 ^c^	17.12 ± 3.02 ^a^	34.71 ± 2.38 ^cde^	35.49 ± 1.06 ^bcd^	63.51 ± 0.02 ^bcde^	64.59 ± 0.05 ^b^
50	28.19 ± 1.69 ^abc^	24.77 ± 2.84 ^cd^	13.39 ± 0.63 ^bc^	15.02 ± 2.01 ^abc^	36.83 ± 2.02 ^bc^	32.59 ± 2.63 ^ef^	62.43 ± 0.25 ^efgh^	59.94 ± 0.32 ^i^
60	28.58 ± 3.34 ^ab^	28.72 ± 4.09 ^ab^	12.91 ± 0.14 ^bc^	12.73 ± 2.16 ^c^	31.42 ± 0.18 ^f^	33.69 ± 2.83 ^def^	62.26 ± 0.56 ^efgh^	61.20 ± 1.10 ^hi^

Note: statistics are shown as mean ± standard deviation; means bearing different superscripts under the same properties show significant difference at *p* < 0.05.

**Table 3:** Effect of frying methods and cycles on the formation of TFAs (mg/g) in duck skin and breast meat.

Sample	Number of Cycles	*9t*C16:1		*t*C18:1	
		Traditional Oil Frying	Water–Oil Mixed Frying	Traditional Oil Frying	Water–Oil Mixed Frying
Skin	0	0.19 ± 0.01 ^c^	0.18 ± 0.01 ^cd^	0.90 ± 0.00 ^g^	0.92 ± 0.02 ^fg^
	1	0.17 ± 0.01 ^ef^	0.16 ± 0.02 ^ef^	1.07 ± 0.11 ^defg^	1.01 ± 0.25 ^efg^
	10	0.17 ± 0.01 ^de^	0.18 ± 0.00 ^cd^	1.08 ± 0.05 ^cdef^	1.11 ± 0.01 ^bcde^
	20	0.17 ± 0.01 ^ef^	0.18 ± 0.00 ^cd^	1.07 ± 0.04 ^def^	1.06 ± 0.04 ^defg^
	30	0.16 ± 0.01 ^ef^	0.17 ± 0.01 ^ef^	1.05 ± 0.03 ^defg^	1.10 ± 0.10 ^cde^
	40	0.18 ± 0.00 ^cd^	0.16 ± 0.00 ^f^	1.20 ± 0.07 ^abcd^	1.20 ± 0.02 ^abcd^
	50	0.20 ± 0.00 ^b^	0.19 ± 0.02 ^c^	1.25 ± 0.13 ^abc^	1.28 ± 0.02 ^ab^
	60	0.22 ± 0.01 ^a^	0.20 ± 0.00 ^b^	1.31 ± 0.22 ^a^	1.29 ± 0.07 ^a^
Meat	0	ND	ND	0.07 ± 0.01 ^g^	0.07 ± 0.01 ^g^
	1	ND	ND	0.08 ± 0.01 ^de^	0.09 ± 0.01 ^d^
	10	ND	ND	0.09 ± 0.00 ^cd^	0.08 ± 0.01 ^de^
	20	ND	ND	0.07 ± 0.00 ^fg^	0.08 ± 0.00 ^def^
	30	ND	ND	0.07 ± 0.01 ^fg^	0.07 ± 0.02 ^efg^
	40	ND	ND	0.10 ± 0.00 ^bc^	0.11 ± 0.01 ^ab^
	50	ND	ND	0.08 ± 0.00 ^def^	0.12 ± 0.01 ^a^
	60	ND	ND	0.09 ± 0.00 ^cd^	0.10 ± 0.01 ^b^

Note: statistics are shown as mean ± standard deviation; means bearing different superscripts under the same properties show significant difference at *p* < 0.05. ND = Not detected

**Table 4:** Effect of frying methods and cycles on the formation of HCAs (ng/g) in duck skin and breast meat.

Sample	No. of Cycles	Norharman		Harman		PhIP		AαC		MeAαC		Total	
		Traditional Oil Frying	Water–Oil Mixed Frying	Traditional Oil Frying	Water–Oil Mixed Frying	Traditional Oil Frying	Water–Oil Mixed Frying	Traditional Oil Frying	Water–Oil Mixed Frying	Traditional Oil Frying	Water–Oil Mixed Frying	Traditional Oil Frying	Water–Oil Mixed Frying
Skin	0	ND	ND	ND	ND	ND	ND	ND	ND	ND	ND	ND	ND
	1	0.25 ± 0.02 ^f^	0.28 ± 0.01 ^f^	0.27 ± 0.04 ^cd^	0.32 ± 0.02 ^abcd^	ND	ND	0.09 ± 0.00 ^g^	0.10 ± 0.02 ^g^	ND	ND	0.61	0.70
	10	0.33 ± 0.01 ^e^	0.34 ± 0.00 ^e^	0.28 ± 0.03 ^cd^	0.30 ± 0.01 ^abcd^	ND	ND	0.16 ± 0.01 ^d^	0.08 ± 0.02 ^g^	ND	ND	0.77	0.72
	20	0.39 ± 0.03 ^d^	0.42 ± 0.02 ^bc^	0.30 ± 0.01 ^abcd^	0.26 ± 0.00 ^d^	ND	ND	0.13 ± 0.01 ^ef^	0.15 ± 0.00 ^de^	ND	ND	0.82	0.83
	30	0.34 ± 0.02 ^e^	0.31 ± 0.01 ^e^	0.28 ± 0.12 ^bcd^	0.28 ± 0.04 ^cd^	0.12 ± 0.02 ^e^	0.14 ± 0.03 ^d^	0.08 ± 0.02 ^g^	0.11 ± 0.01 ^f^	ND	ND	0.82	0.84
	40	0.44 ± 0.06 ^bc^	0.40 ± 0.01 ^cd^	0.34 ± 0.03 ^abcd^	0.31 ± 0.02 ^abcd^	0.15 ± 0.01 ^cd^	0.17 ± 0.01 ^ab^	0.11 ± 0.02 ^f^	0.12 ± 0.01 ^f^	ND	ND	1.04	1.00
	50	0.45 ± 0.02 ^b^	0.43 ± 0.01 ^bc^	0.36 ± 0.02 ^ab^	0.34 ± 0.04 ^abc^	0.15 ± 0.01 ^bcd^	0.16 ± 0.01 ^abc^	0.20 ± 0.02 ^b^	0.17 ± 0.01 ^cd^	ND	ND	1.16	1.10
	60	0.49 ± 0.03 ^a^	0.43 ± 0.02 ^bc^	0.37 ± 0.12 ^a^	0.34 ± 0.04 ^abcd^	0.15 ± 0.02 ^cd^	0.17 ± 0.02 ^a^	0.22 ± 0.02 ^a^	0.19 ± 0.01 ^bc^	ND	ND	1.23	1.13
Meat	0	ND	ND	ND	ND	ND	ND	ND	ND	ND	ND	ND	ND
	1	0.47 ± 0.00 ^h^	0.66 ± 0.02 ^ef^	0.59 ± 0.01 ^fg^	0.68 ± 0.08 ^def^	ND	ND	0.09 ± 0.01 ^ef^	0.07 ± 0.03 ^f^	ND	ND	1.15	1.41
	10	0.58 ± 0.05 ^g^	0.73 ± 0.03 ^de^	0.63 ± 0.07 ^ef^	0.70 ± 0.02 ^def^	ND	ND	0.15 ± 0.01 ^abcd^	0.10 ± 0.05 ^ef^	ND	ND	1.36	1.53
	20	0.58 ± 0.04 ^fg^	0.83 ± 0.06 ^bc^	0.51 ± 0.10 ^g^	0.64 ± 0.02 ^ef^	0.13 ± 0.00 ^c^	0.17 ± 0.01 ^a^	0.13 ± 0.00 ^cde^	0.11 ± 0.02 ^def^	ND	ND	1.35	1.75
	30	0.77 ± 0.06 ^cd^	1.00 ± 0.03 ^a^	0.59 ± 0.00 ^fg^	0.67 ± 0.05 ^def^	0.11 ± 0.02 ^d^	0.15 ± 0.02 ^ab^	0.15 ± 0.01 ^bcd^	0.13 ± 0.01 ^cde^	ND	ND	1.62	1.95
	40	0.87 ± 0.07 ^b^	0.99 ± 0.13 ^a^	0.73 ± 0.10 ^bcde^	0.82 ± 0.14 ^ab^	0.12 ± 0.00 ^cd^	0.15 ± 0.01 ^b^	0.18 ± 0.07 ^ab^	0.14 ± 0.01 ^bcde^	ND	ND	1.90	2.10
	50	0.87 ± 0.02 ^b^	0.98 ± 0.05 ^a^	0.71 ± 0.02 ^cde^	0.93 ± 0.14 ^a^	0.12 ± 0.00 ^cd^	0.13 ± 0.01 ^cd^	0.20 ± 0.06 ^a^	0.15 ± 0.01 ^abcd^	ND	ND	1.90	2.19
	60	1.01 ± 0.03 ^a^	0.99 ± 0.05 ^a^	0.76 ± 0.02 ^bcd^	0.81 ± 0.06 ^bc^	0.12 ± 0.00 ^cd^	0.15 ± 0.01 ^b^	0.16 ± 0.05 ^abc^	0.16 ± 0.02 ^abc^	0.03 ± 0.0 ^a^	ND	2.08	2.11

Note: statistics are shown as mean ± standard deviation; means bearing different superscripts under the same properties show significant difference at *p* < 0.05. ND = Not detected.

## Data Availability

Not applicable.
